# West Nile Virus Lineage 2 in Free-Living *Corvus cornix* Birds in Poland

**DOI:** 10.3390/tropicalmed8080417

**Published:** 2023-08-16

**Authors:** Jowita S. Niczyporuk, Wojciech Kozdrun, Agnieszka Czujkowska, Yannick Blanchard, Mariteragi Helle, Nolwenn M. Dheilly, Gaelle Gonzalez

**Affiliations:** 1 National Veterinary Research Institute Pulawy (NVRI), Department of Poultry Diseases, Al. Partyzantow 57, 24-100 Pulawy, Poland; 2 Rehabilitation Center for Protected National Birds “Bird Asylum”, Av. Ratuszowa 1/3, 03-461 Warszawa, Poland; a.czujkowska@zoo.waw.pl; 3 Génétique Virale et Biosécurité, French Agency for Food, Environmental and Occupational Health & Safety (ANSES), Laboratoire de Ploufragan-Plouzané-Niort, Université de Rennes 1, 22440 Ploufragan, France; yannick.blanchard@anses.fr; 4 ANSES, INRAE, Ecole Nationale Vétérinaire d’Alfort, UMR VIROLOGIE, Laboratoire de Santé Animale, 14 rue Pierre et Marie Curie, 94701 Maisons-Alfort, Francenolwenn.dheilly@anses.fr (N.M.D.);

**Keywords:** crow, detection, RT-PCR, (q) RT-PCR, wild birds, zoonoses

## Abstract

The summer temperatures recorded in Poland in 2022 were among the highest in over 30 years and, combined with higher-than-expected rainfall, gave the impression of an almost tropical climate. Such climatic conditions were ideal for the transmission of vector-borne zoonotic diseases such as West Nile fever. In northeastern Poland, in the Mazowieckie region, the Polish event-based surveillance network reported increased fatalities of free-living hooded crows (*Corvus corone cornix)*. West Nile virus (WNV) lineage 2 was identified for the first time as the etiological agent responsible for the death of the birds. WNV was detected in 17 out of the 99 (17.17%) free-living birds tested in this study. All the WNV-infected dead birds were collected in the same area and were diagnosed in September by the NVRI and confirmed by the EURL for equine diseases, ANSES, in October 2022. Unnaturally high temperatures recorded in Poland in 2022 likely favored the infection and spread of the virus in the avian population. A nationwide alert and awareness raising of blood transfusion centers and hospitals was carried out to prevent human infections by WNV.

## 1. Introduction

West Nile virus, an enveloped RNA arbovirus, is a member of the *Flaviviridae* family, genus *Flavivirus*, which circulates in an enzootic transmission cycle between mosquitoes mainly from the *Culex* genus and wild bird species as an amplifying host, by mortality and viremia, and wild bird species consequently play a relevant role as reservoirs [1,2]. Humans, equines, and other mammals are dead-end hosts, unable to infect a naïve mosquito during a blood meal [3,4,5]. WNV was first isolated from the blood of a febrile elderly woman in the West Nile district in Uganda in 1937 [6] and has spread around the world since that time [7]. WNV infection is mainly subclinical or asymptomatic. In less than 20% of cases, people experience a mild flu-like illness, and less than 1% of them will develop neuroinvasive diseases such as encephalitis and meningitis. Sequencing and phylogenetic analyses have confirmed nine phylogenetic lineages of WNV circulating worldwide, but only WNV lineages 1 and 2 circulate in central Europe and have been responsible for epidemics in humans and equines [8,9].

Free-living predatory birds remain a perfect reservoir for a number of viruses that are commonly contagious and fatal for domestic birds but persist in the subclinical stage without any signs of clinical disease. WNV infections have been documented in many taxonomic groups of captive and wild birds [2], such as corvids and raptors, but few data are available on WNV infection in waterfowl species, such as mallards (*Anas platyrhynchos*) [10]. As evidence, the circulation of WNV between free-living resident and migratory birds was also confirmed [11]. WNV-neutralizing antibodies have been detected less frequently in Serbia among resident birds than migratory birds, which may suggest their significant role as reservoirs of the virus. In contrast, resident birds may act as amplifiers of local WNV strains. It is well accepted that wild migratory birds are able to transfer arboviruses during their migrations (flights/migration to remote areas from their place of existence). Therefore, they could facilitate the spread of the virus in new geographic areas [12].

Poland is located within central migration routes between Africa, Europe, and Asia and is home to many bird species as well as an important migratory stop for birds. Climate change could alter the migration corridors as well as the life cycle of *Culex* mosquitos, such as *Culex modestus* [13] and *Culex pipiens* [14,15], causing them to develop in new areas/niches. WNV can adapt to new ecological niches and expand its distribution in new areas where it was absent before [1,16]. Such a situation took place in the 2000s in Europe, and since then, the virus has spread throughout central Europe, affecting more and more countries every year [17]. Even though neighboring countries have reported WNV infection in wild birds, WNV outbreaks have not been reported in Poland, with only indirect evidence indicating the circulation of WNV-like viruses in migratory birds. Positive results of serological tests carried out in wild birds showed the presence of specific antibodies IgM against WNV in Poland [18,19,20]. In Poland, we run an active and passive surveillance system as part of a research program on wild avifauna. We expect to detect WNV seroconversion in the sera of resident birds or WNV genomic RNA in carcasses of unexplained dead birds as in other countries as presented by Chancey in 2015. The implementation of such an active and passive surveillance system is worth operating in Poland especially during this period of rapid climate change and the rapid expansion of WNV lineage 2 in countries in western and northern Europe [20,21] such as Germany [21] and the Netherlands [22].

During the 2022 transmission season, between 11 September and 28 October 2022, over 99 fatal cases among free-living birds belonging to *Anseriformes*, *Passeriformes*, *Strigiformes*, *Falconiformes*, *Ciconiiformes*, and *Accipitriformes* orders were reported by the Polish event-based surveillance system. We expected transmission in autumn, connected with the life cycle of mosquitoes of the genus *Culex*, which are the main vector of the virus, and with the migration of birds, which are the main reservoir of the virus.

The aim of this study was to confirm whether the deaths of these birds were related to viral infection and especially to the West Nile virus. We also evaluated the available data concerning WNV infection in free-living birds in Poland.

## 2. Materials and Methods

### 2.1. Study Region

**Figure 1:**
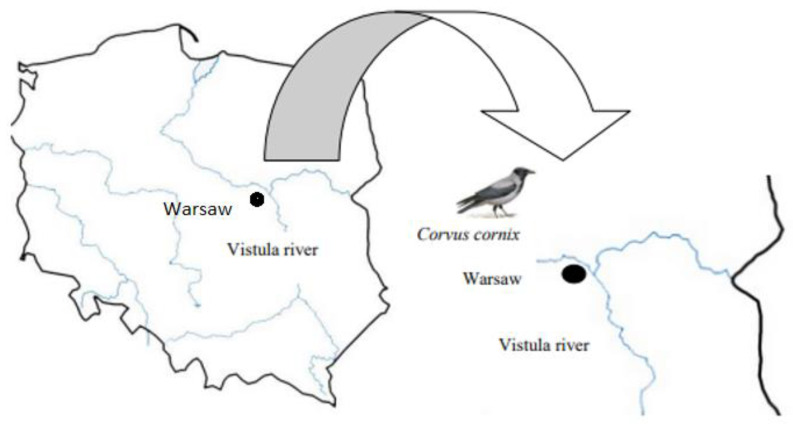
Infected free-living bird area in 2022, Warsaw, Poland. Holding with WNV-positive cases is marked with black dot. Defined nature reserve in the Central Mazowieckie Lowlands.

The studies were conducted in Warsaw (φ 52°13′ N, λ 21°00′ E), a region in the central part of the largest city in the country and in the Central Mazowieckie Lowlands, on the Vistula River, Poland (Figure 1). The climate of Poland is a transitional climate between oceanic and continental. This is reflected in the diversity of climatic elements. The average annual rainfall is 600 mm. The lowest rainfall is found in Kujawy and eastern Wielkopolska—below 500 mm. The heaviest precipitation is found in the mountainous areas (the Sudetes and Carpathians with over 1000 mm, the Tatras—1700 mm).

### 2.2. Ethical Statement

All samples tested in the studies were obtained from the “Bird Asylum” Rehabilitation Centre for Protected National Birds (ptasiazyl.zoo.waw.pl) and from official veterinarians following handling procedures implemented following European (86/609) and Polish laws (Special edition in Polish: Chapter 15, Volume 001, pp. 292–320) with the aim to reduce the suffering of animals. The research in the above manuscript was performed on the carcasses of dead birds in accordance with the rules of ethics.

### 2.3. Sampling Collection

From September 2022 to October 2022, 99 free-living birds belonging to 6 different orders represented by 10 avian species were collected in collaboration with the “Bird Asylum” Rehabilitation Centre for Protected National Birds and official veterinarians. Brain samples were collected during the necropsy of each individual bird. Among the 99 wild birds, 21 presented with neurological signs such as tremors, unnatural behavior with abnormal body posture, seizures, and paralysis, as reported by the veterinarians of the Rehabilitation Centre for Protected National Birds and official veterinarians.

### 2.4. RNA Extraction

Viral RNA was extracted from 200 µL of brain homogenates using the viral RNA Mini Kit (Qiagen, Hilden, Germany) following the manufacturer’s protocol. Viral RNA was suspended in an RNase inhibitor buffer (Life Science, Morrill Hall, IL, USA), and concentrations were determined using a spectrophotometer (Biorad, Hercules, CA, USA). Viral RNA was kept at −80 °C before use.

### 2.5. Control Samples

The WNV infectious strain New-York 1999 (GenBank accession: AF196835) isolated from the brain of an infected horse was kindly provided by the European Union Reference Laboratory for WNV studies (ANSES Maisons-Alfort, France). Two negative controls were used during RT-PCR and were acquired from chicken embryo fibroblast (CEF) and SPF cultures.

### 2.6. Primers and RT-PCR Amplification of WNV

Reverse transcription RT-PCR primers were designed to target the conserved 3′NCR sequence (non-coding region, GeneBank accession number: DQ211652; one primer set, a forward and reverse primer), and the sequences were as follows: WNVF (sense primer): 5′ AAA GCC CAA TGT CAG ACC AC 3′ and antisense primer WNVR: 5′ TAG TCC TTT CGC CCT GGT TA 3′, as indicated and developed by Niczyporuk, 2011.

### 2.7. Molecular Studies

The RT-PCR method described by [18] was employed to detect the WNV genome. (q) RT-PCR was then conducted, following the protocol for the molecular detection method of WNV described by [23,24], and the viral genome load was confirmed by (q) RT-PCR by ANSES EURL for West Nile virus.

### 2.8. Full-Length WNV Genome Sequencing—Sequencing Analysis

Sequencing libraries were prepared from two pools of genomic RNAs extracted from brain samples/homogenates. Whole genome WNV sequences were obtained using the Illumina High Seq technology (Life Technologies, Carlsbad, CA, USA) as previously described by [25] and assembled with BWA Mapper for GenBank accession number LR743458.1, 2019. We compared the assembled sequences from each pool, and they were identical. We inputted the IUPAC code (Y, R K) when read alignments revealed the presence of a polymorphism in the viral population that existed in both pools. The full-length WNV genome sequence was extracted by mapping all readings against the new viral reference genome (determined following a blast of de novo contigs) using BWA (version 0.7.8). Sequences obtained as part of this study are available from GenBank under accession number PL-0P804520.

### 2.9. Phylogenetic Analysis

The obtained West Nile virus sequence was compared with West Nile virus sequences obtained from the GenBank (NCBI) database. The analysis was conducted based on nucleotide sequences of the whole genome sequences using the maximum likelihood method and the Tamura–Nei substitution model in Mega 11 software with 1000 bootstrap replications. The evolutionary distances were computed using the maximum composite likelihood method [26] and are in the units of the number of base substitutions per site. Ancestral states were inferred using the maximum likelihood method [26] and the Tamura–Nei model [27]. The tree shows a set of possible nucleotides (states) at each ancestral node based on their inferred likelihood at site 1. Bio-algorithms for a matrix of pairwise distances were estimated using the Tamura–Nei model, and then the topology with a superior log-likelihood value was selected. The rates among sites were treated as being uniform among sites (uniform rate option). This analysis involved 35 nucleotide sequences. There were a total of 11,017 positions in the final dataset. Evolutionary analyses were conducted in MEGA 11 [28].

## 3. Results

### 3.1. Studies

The Polish event-based surveillance system reported massive mortality (99 individuals) of wild birds, in September–October 2022, belonging to the following avian orders/species: *Anseriformes* (*Cygnus olor* and *Anas platyrhynchos*), *Passeriformes* (*Corvus cornix*, *Corvus frugilegus*, and *Corvus monedula)*, *Strigiformes* (*Strix aluco* and *Bubo scandiacus*), *Falconiformes* (*Falco peregrinus*), *Ciconiiformes* (*Ciconia Ciconia*), and *Accipitriformes* (*Buteo buteo*). The largest group (n = 49) was constituted of birds from *Passeriformes (Corvus cornix)*, while the least frequently diagnosed was from *Corvus frugilegus* (n = 2). Detailed information concerning bird species is indicated in Table 1. The brain samples obtained from the 99 birds, from which 21 birds manifested neurological signs of infection, were subjected to molecular detection for *flaviviruses*.

### 3.2. RT-PCR and Real-Time RT-PCR

Samples were analyzed by RT-PCR to detect and identify RNA sequences characteristic of the West Nile virus by the method obtained from Niczyporuk, 2011. Seventeen hooded crows (*Corvus cornix*) collected in September 2022 were positive for WNV *flavivirus* from the JE serocomplex. The samples with positive results were confirmed by EURL ANSES using (q real-time) RT-PCR, and the cycle threshold (Ct) values of the 17 brain samples ranged from 15.38 to 27.237 (mean 20.12). Seventeen of them were found to be infected with WNV.

### 3.3. Sequencing Analysis

Seventeen positive amplicons were obtained and sequenced. In this original research, genome sequences of WNV Crow 1-17-Pulawy-PL (lineage 2) were used in accordance with the protocols conducted by EURL ANSES using BWA (version 0.7.8). The sequences of the same strain were obtained in all 17 samples; therefore, the entire sequence was submitted to the NCBI GenBank database under the accession number OP804520.

### 3.4. Phylogenetic Tree

**Figure 2:**
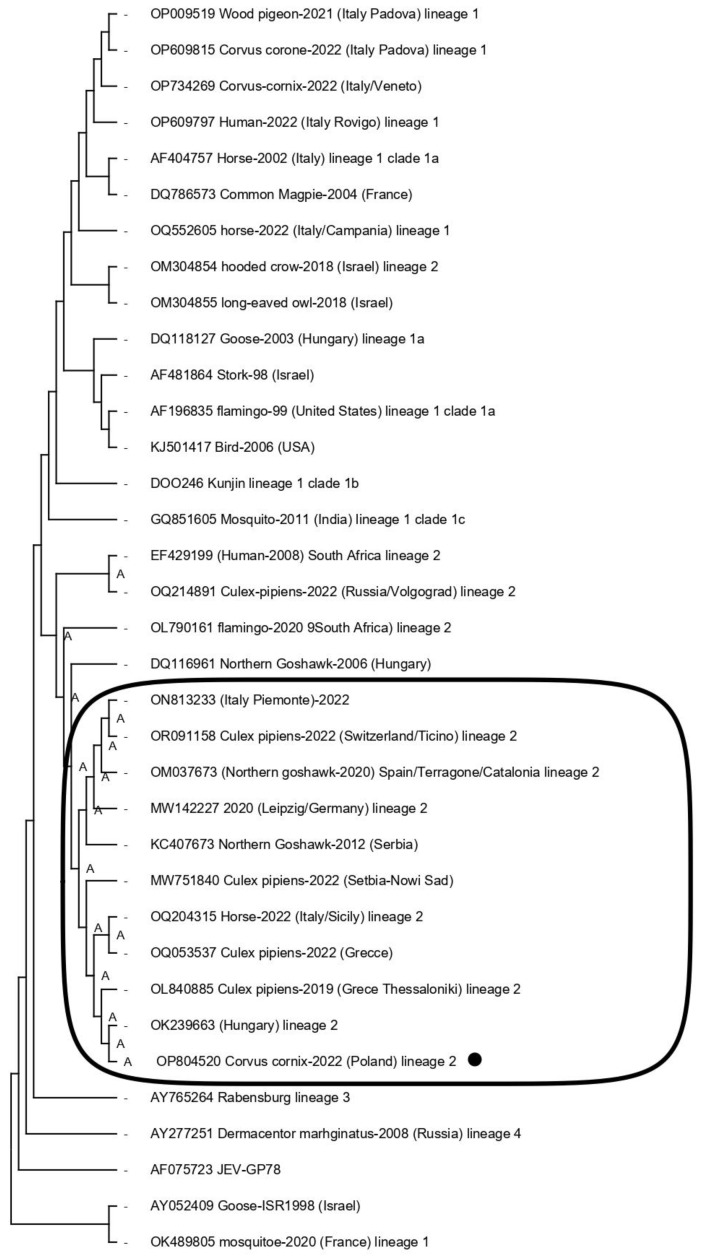
Phylogenetic analysis of a WNV strain sequence from avian species (*Corvus cornix*) studied in Poland and compared with obtained WNV sequences from other countries/continents representing different WNV lineages (Lin 1–9) deposited in the GenBank NCBI database.The frame shows the sequences of strains classified as lineage 2 of WNV circulating in Central Europe, which are arranged in one characteristic group, showing close similarity. In addition, the black dot indicates the sequence of the WNV strain that was marked in Poland for the first time.

A phylogenetic tree was constructed by aligning the nucleotide sequences of the amplified fragments from the WNV genome. All ambiguous positions were removed for each sequence pair (pairwise deletion option). There were a total of 11,017 positions in the final dataset. The phylogenetic tree demonstrated WNV lineage 2 detected in Poland for the first time (GenBank accession No. OP804520 *Corvus cornix av1-17/22,* Figure 2). These sequences clearly positioned themselves on the tree, creating a separate group of strains representing the WNV lineage 2, having their ancestors in the South Africa strain (OL790161) isolated from flamingo in 2020 and the strain OK239663 from Hungary. As a result of deep analyses, the Polish sequence turned out to be similar to the sequences isolated in 2022; it forms one clear group of strains in which we can find two strain sequences (ON813233 and OQ204315) from Italy, also isolated in 2022, one strain sequence (OR091158) from Switzerland in 2022, one sequence (OM037673) from Spain in 2020, a sequence (MW142227) from Germany in 2020, a sequence (MW751840) from Serbia/Nowi Sad in 2022, and two sequences (OL840885 and OQ053537) from Greece in 2019 and in 2022, respectively. The molecular characterization of the Polish WNV strain was carried out. Viral genome sequence identities of the WNV strain were in the range of 99.8% at the nucleotide (nt) and amino acid (aa) levels. Phylogenetic analysis has shown that these WNV strains are currently circulating in central Europe.

## 4. Discussion

WNV infections were pointed out for the first time in wild birds (*Corvus cornix)* in Poland, indicating the emergence of the virus in Mazowieckie Voivodeship and the Central Mazowieckie Lowlands, on the Vistula River. The viral strain sequenced in each location is genetically similar to WNV lineage 2 strains isolated in central Europe, in particular in Hungary in 2018. Our findings indicate that the Polish strain is pathogenic in birds even if WNV infections in wild avifauna are difficult to quantify and detect by WNV-specific surveillance programs.

From the southern mountain ranges to the Baltic Sea coast, Poland contains some of the most diverse ecosystems in Europe, with a corresponding wealth of flora and fauna. The extraordinarily intricate topography of Poland provides a healthy habitat for resident and wild avifauna. The Poland bird checklist indexes 467 bird species belonging to 27 families wintering in Poland on 31 December 2022 (according to the division adopted by the Faunistics Commission of the Ornithological Section of the Polish Zoological Society). Birds belonging to Corvidae are described as a reservoir for WNV in Europe [29,30]. This highlights the need to extend the avifauna surveillance program in Poland in order to detect early WNV circulation in the country. WNV circulation has already been identified indirectly by sero-surveillance in 2014 and 2017 in 62 storks, 1 common chaffinch (*Fringilla coelebs*), 1 horse, and 14 humans [19,20], as well as in 2022 in a northern goshawk (*Accipiter gentilis*) suspected for WNV infection (data not published). WNV circulation expansion in central, western, and northern Europe is expected to continue in the near future thanks to climatic and environmental conditions threatening Europe [31].

The current study indicates the presence/circulation of WNV strains in *Corvus cornix* in Poland. This analysis is extremely important information for further epidemiological analyses of wild birds in the region. The spread of the virus is dependent on ornithophilic mosquitoes (the main virus vector) and wild bird species (the reservoir of the virus). These are the links in the chain of the epidemiology of this virus. Molecular analysis of the 2022 WNV outbreak in Poland indicated 17 positive WNV fatalities in *Corvus cornix*, confirming WNV lineage 2 infection in Poland for the first time. From an epidemiological point of view, wild birds represent a source of multiple pathogens and various infectious diseases. In this study, the infected birds belonged to resident species. Most important from an epidemiological point of view were the birds belonging to *Passeriformes*. They are the primary birds involved as a reservoir of the virus and are also sensitive to WNV infection [30].

A very large role in the spread of the virus in the world is attributed to the local movement of sedentary bird species and the long journeys of migrating birds, which cover tens of thousands of kilometers between breeding and wintering grounds [9,12]. Antibodies to West Nile virus have been detected in 326 species of birds, but not all of them can equally act as reservoirs of the virus. The most important bird species in the life cycle of WNV are presented below. Exotic wetland birds are the main reservoir of the virus in tropical countries. The potential main reservoirs and hosts of the virus in Poland are the white stork and Corvidae families. Therefore, the reservoir of the virus is species of sedentary birds, as well as migrating birds [9,14,17,31]. The most important bird species that are the WNV reservoir in Poland are the white stork, Eurasian chaffinch, mute swan, and grey crow [19,20].

The confirmation of WNV infection in *Passeriformes* (*Corvus cornix*), the most common positive species in the studies, has also been indicated in 2022 in Israel [30] and Italy [32].

In Europe, WNV in wild resident and migratory birds in Serbia with 8% of confirmed cases with specific WNV antibody detection in four mute swans, two white-tailed eagles, and one common pheasant [1] suggests that migratory birds can act as reservoirs of WNV. Positive serological results in wild birds and horses have also been obtained in Germany [32,33] and Serbia (eCDC, 2022). However, in 2018, WNV was detected and confirmed in eastern and southeastern Germany for the first time in resident and wild common blackbirds (*Turdus merula*), northern goshawks (*Accipiter gentilis*), and great grey owls (*Strix nebulosa*) [21]. Current studies on free-living birds in Poland have shown positivity for WNV antibodies [19,20]. The current trends in the spread of WNV are expected to continue in the future and transmit to different countries in which the virus never previously existed. This is especially concerning, considering the high impact of climate change [31]. Along with the cases from Poland, since the beginning of the 2022 transmission season, 314 outbreaks among birds have been reported in Italy (249), Germany (51), Spain (9), Austria (2), Croatia (2), and Hungary (1) [34]. The phylogenetic analysis in the conducted studies indicated that all 17 WNV strains that were collected from *Corvus cornix* species in September 2022 were classified as lineage 2 and cluster 2 in eastern Europe. The sequences obtained in September 2022 in Poland were closely related to several WNV strain sequences reported previously in outbreaks in 2022 in Europe: an outbreak in Italy/Piemonte and Italy/Sicily, lineage 2; a strain sequence isolated from Greece (OQ053537 and MW751840) and Serbia/Nowi Sad, 2022; and a strain sequence from goshawk isolated in Serbia in 2012 (KC407673) and Hungary in 2006 (DQ116961). We can see that this strain is being introduced globally (sequences isolated in Greece, Hungary, Italy, and Spain are closely related and probably depend on the same virus reservoir—free-living wild bird species and climate conditions (warm and windy summer in 2022 in Poland)) and that this epidemiology is developing.

This contagious virus is very dangerous for birds, and especially for geese as was confirmed and presented in 1997 in Israel, with morbidity ranging from 20% to 60%. Circulation of WNV in Hungary has been known since 1969 [35] when the presence of the first virus strain was isolated and confirmed. In 2003, a WNV outbreak was confirmed in Hungary, with the morbidity and mortality reaching about 14%, and in Romania during WNV outbreaks [14]. The virus is circulating in wild birds in southern France [36], in birds of prey and predatory birds in Hungary [37], in the eastern part of Austria [38], and in the wild Eurasian magpie (*Pica pica*) in Greece [39]. Infected birds have been confirmed in Serbia, including northern goshawks (*Accipiter gentilis*), white-tailed-eagles *(Haliaeetus albicilla),* legged gulls (*larus michahellis*), hooded crows (*Corvus cornix*), bearded parrotbills (*Panurus biarmicus*), and common pheasants (*Phasianus colchicus*) [11], and infections have been confirmed in resident birds in southern France [12] and in griffon vultures (*Gyps fulvus*) and little owls (*Athene noctua*) in Spain [40].

The understanding of all factors, namely the epidemiological, ecological, and climatic conditions (temperatures and periods with heavy rainfall and low rainfall) together with evolutionary processes and spreading of the arboviral disease, is extremely critical and can lead us to better understand and to develop surveillance strategies to control the virus.

The risk of West Nile transmission depends on the environmental temperature. From year to year, we have witnessed an increase in the temperature and humidity rates as well as early falls reported in Europe. Poland is particularly exposed to the occurrence of re-emerging pathogens, such as WNV that may be endemic in specific regions such as the ones screened in our study. From year to year, the temperature and humidity in summer and early fall are increasing and maintaining an upward trend [35]. From year to year in our climate, we are also exposed to the occurrence of re-emerging pathogens, such as WNV. However, the first isolation of WNV from *Corvus cornix* was in Poland. The *Corvidae* family belongs to the group of bird species most susceptible to infection with the West Nile virus and, at the same time, is considered to be the most important reservoir of the virus with high levels of viremia and massive shedding of virus particles through cloacal and oral fluids. Positive results in these species suggest that the virus may become localized because the population of these birds is significant and the risk of WNV transmission has increased. The EURL for WNV (ANSES, Maisons-Alfort) confirmed the diagnosis. Phylogenetic analysis of the two WNV RNA genomes demonstrated that they were identical and arose as the same introduction event. WNV isolated from Poland was closely related to the WNV/Hungarian strain, OK239663 lineage 2, with 99.8% identity (Figure 2).

## 5. Conclusions

During studies, only hooded crows were positive for WNV infection, and this species is the most susceptible to infection and the most frequently recorded as infected in the world.

Poland faced its first outbreak of WNV infection in wild avifauna (*Corvus cornix)* during the 2022 transmission season. This study presents evidence for the presence of WNV infection in the northeastern wild bird population in Poland. The presence of genetic material of WNV demonstrates that this virus can circulate in this particular study area among wild birds in the 2023 season.

We provided the first sequence of the WNV strain circulating in the country. It belongs to the European WNV lineage 2 (GenBank accession number: PL-0P804520). Lineages 1 and 2 are the most important from a zoonotic side of infection and are isolated from many species, e.g., birds, humans, and horses.

This emphasizes the need to implement a surveillance program in order to identify the other components of WNV circulation, especially the equine and human populations, to protect the elderly in particular. The objective is to deploy an early warning system ahead of a possible introduction of WNV into new geographic areas in Poland.

## Figures and Tables

**Table 1:** Results obtained from the RT-PCR molecular diagnostic method for detection of West Nile virus in free-living birds by order/species indication conducted in Poland in the 2022 season with positive results obtained for *Passeriformes (Corvus cornix)*.

Avian Order	Avian Species	Analysed (n)	Result
*Anseriformes* (Waterfowl)	*Cygnus olor* *Anas platyrhynchos*	12 5	Negative Negative
*Passeriformes* (Corvidae)	*Corvus cornix* *Corvus frugilegus* *Corvus monedula*	49 4 2	Positive 17 (34.69%) Negative Negative
*Strigiformes* (Owls)	*Strix aluco* *Bubo scandiacus*	1 1	Negative Negative
*Falconiformes* (Falconidae)	*Falco peregrinus*	3	Negative
*Ciconiiformes* (Storks)	*Ciconia ciconia*	18	Negative
		99	Positive 17 (17.17%)

## Data Availability

Not applicable.
